# Successful Laparotomy for Gunshot Wound of Intestines

**Published:** 1885-09

**Authors:** 


### Cullings From Contemporaries.

### GUNSHOT WOUND OF INTESTINES—LAPAROTOMY,—RECOVERY.

Prof. J. B. Hamilton, Surgeon General M. H. S., gives, in the Jour. Am. Med. Ass., the details of a case of pistol shot wound of the abdomen (colored man, aged 19), where the bullet, a 32 calibre, passed through several coils of the small intestine and into the ascending colon; wounding, also, the omentum in several places. Dr. H. opened the abdomen, and sewed up, with fine carbolized catgut, eleven wounds in the small intestine and two in the ascending colon, besides ligating and removing a wounded mass of the omentum; dressed the wound according to the latest antiseptic practice, and gave opium freely. The progress of the case was characterized by much tympanitis, an exhausting diarrhœa, and great tenesmus. The bullet passed from the bowels at stool, on the thirteenth day. The tenesmus was due to a large pelvic hæmatoma, supposed to have been caused by continued oozing from some minute wound overlooked or not ligated. The hæmatoma was punctured through the rectum, and discharged first, a pint and a half of thin, offensive blood, and on another occasion, during an attempt to break down and discharge the blood clot, another gush came of about the same amount. Becoming anxious, the surgeon plugged the rectum with lint above, and ice below the puncture, and prevented further hemorrhage. The patient recovered, and was dismissed August 8, twenty-eight days after the receipt of the injury.

There can be no doubt that this man would have died a violent death of peritonitis and septicæmia within the first week under the old let-alone policy.

This, we believe, constitutes the second case on record of successful laparotomy for wounded intestines—at least in this country, and in man—Dr. Bull's case being the first (New York *Medical Journal*, Feb. 24, 1885).

Prof. Charles Parke, of Chicago, made several successful laparotomies for gunshot wound of the intestines of dogs—but men are not dogs—most of them. We saw one, Dr. Parke exhibited at the Washington meeting, on which he had operated for such wounds. He was exceedingly lively, but entirely dumb—couldn't bark.

